# Animal-Assisted Therapy in the Residential Treatment of Dual Pathology

**DOI:** 10.3390/ijerph17010120

**Published:** 2019-12-23

**Authors:** Miguel Monfort Montolio, Javier Sancho-Pelluz

**Affiliations:** 1 Doctorate School, Universidad Católica de Valencia San Vicente Mártir, 46001 Valencia, Spain; 2 Amigo Foundation, 12006 Castellón, Spain; 3 Neurophysiology and neurobiology, School of Medicine and Health Sciences, Universidad Católica de Valencia San Vicente Mártir, 46001 Valencia, Spain; fj.sancho@ucv.es

**Keywords:** animal-assisted therapy, life skills, impulsiveness, residential treatment, dual pathology

## Abstract

Animal-assisted therapy (AAT) is a complementary intervention of therapy that has shown positive results in the treatment of various pathologies. This study assesses the viability of the implementation and the effectiveness of an AAT program in patients diagnosed with substance abuse disorder and associated mental disorders (dual pathology). For the study, a dynamic prospective cohort was used, consisting of 43 patients in residential treatment. The program consisted of 10 sessions with a duration of about 60 min, where data was collected in the 3rd, 6th and 10th sessions. The Life Skills Profile questionnaire (LSP) and the Barratt Impulsiveness Scale (BIS-11) were used for subsequent evaluation. Patients who participated in the program showed an improvement in daily skills, which favoured a better quality of life and decreased impulsiveness, enabling them to regain self-control. These results suggest that the dog can be a multi-sensory stimulus that captures attention, and improves motivation, cooperation and patient involvement in therapy. It was concluded that AAT can serve as an adjunctive therapy in the rehabilitation processes of people diagnosed with dual pathology.

## 1. Introduction

Finding the origin of animal-assisted therapy (AAT) is not an easy task, since throughout history it has been supposed that interactions with animals may have benefits to human beings for a variety of reasons. The genesis of AAT goes back to the Neolithic period, with the emergence of the domestication process that significantly transformed the relations of human society with nature [1]. Originally, the interaction between humans and other animals was limited to cohabitation and rivalry for daily food. As human–animal coexistence advanced, it evolved towards the role of humans as the domesticator, a quality that indicated greater social and economic development, because man fed and was served by animals [2].

The coexistence and creation of emotional ties of humans with various tamed animals consolidated in this stage, and the therapeutic benefits that animals provided to humans progressively manifested [3]. Thus, in the 5th century BC, horse riding exercise was used for the rehabilitation of wounded soldiers [4], and the belief that dogs could heal wounds persisted until well after Christianity arrived in Europe [5]. *Galen* also argued that the practice of riding improved rational thinking and that the practice of sports with animals decreased episodes of hypochondria and hysteria [4]. In the Enlightenment period, new theories were introduced highlighting the socializing influence of interaction with animals [6]. At the York Retreat hospital, England, therapeutic contact was introduced for people diagnosed with mental illnesses with small farm animals, in order to promote self-control. In the 19th century, animals such as cats, birds, squirrels, dogs and fish were included in the Bethlem psychiatric hospital as items of therapy [5]. In the 20th century, AAT was applied in the US for the treatment of hospitalized wounded adults and children, with the therapeutic purpose of improving their quality of life [5,7].

Therapeutic interventions that are complemented by AAT provide well-being and promote the health of patients [8]. AAT is an intervention that seeks to achieve a specific objective through participation and interaction with an animal [2]. AAT programs are planned to encourage the improvement of physical, social, emotional and cognitive human functioning. They can be implemented individually or in groups, and the development of the program is always evaluated and documented [9]. One of the main benefits of AAT is the interaction with the animal as a space where the patient’s therapeutic relationship and adherence to the treatment is improved. An emotional and protected environment is promoted through the animals for the development of therapeutic programs [10,11] in order to achieve rehabilitation [12]. Nevertheless, presenting AAT as a therapy in itself is a mistake. AAT features among treatment alternatives, always as a complement to prototypical psychotherapeutic and pharmacological interventions [13]. It is not conceived as a sole treatment but as a complementary option that facilitates the therapist’s work [14]. The animal is used as an instrument that helps, through interaction with the patient, to achieve the therapeutic objectives of the treatment, promoting various activities to address rehabilitation targets such as cognitive, emotional–affective, social or linguistic improvement [15].

AAT can be performed with various animals, be they dogs, cats or other pets, or larger animals such as horses and dolphins [16]. It should be noted that dogs are the most commonly used because of the diversity of their breeds and their ability to be trained [7], encouraging communication and acting as a positive reinforcement for patients [8]. The main environments for intervention are neuro-rehabilitation, education, mental health, geriatrics, gerontology and prison or hospital contexts [17].

Intervention in substance abuse disorders is characterized by the use of various techniques to achieve patient rehabilitation. There is an additional difficulty in achieving this, in that substance abuse is often associated with various negative aspects of patient health [18]. Dual pathology is defined as the presence of a mental disorder and a substance abuse disorder in the same patient [19]. This comorbidity has important implications at clinical and therapeutic levels [20], resulting in poorer prognosis and therapeutic outcome and increased likelihood of relapse in mental or addictive diseases [21]. AAT is used as a complementary therapy in tackling dual pathology [22]. In this therapy, animals are used as therapeutic instruments to deliver various objectives such as enhancing creativity, emotional management, empathy, self-control, social skills training, self-esteem, concentration, and reduction of stress and anxiety and aggressive behaviours through confronting the consequences and working on internal processes that facilitate the assumption of new behaviour [23]. Intervention in dual pathology through AAT helps to overcome one of the main difficulties the therapist has, namely the sustaining of a therapeutic alliance [24]. AAT helps patients become involved and participate in a more active way in their own therapy process. When an animal is included in the treatment, it helps introspection and internalization, increases confidence in the therapeutic environment, provides security and confidence in therapy, and motivates the patient to share their feelings [2,25].

One of the contexts where all these aspects can be observed, while at the same time attempting intervention, is the residential environment, specifically the Therapeutic Community (TC). There has been an increase in TC patients with dual diagnosis in recent years [26,27]. This residential therapeutic–educational scenario maintains the traditional intervention techniques focused on the rehabilitative/psychosocial context, while, at the same time, it has been shown to be a suitable framework for the application of an AAT program with a view to reducing psychopathology, and improving quality of life and adherence to treatment, because the patients enjoy a certain level of clinical permanence with it [28].

The objectives of this study were (a) to demonstrate the viability of the implementation and (b) to evaluate the effectiveness of an AAT program in patients diagnosed with substance abuse disorder and associated mental disorders (dual diagnosis). For the evaluation of the effectiveness of AAT, we take into account the impulsivity and life skills of patients in a residential context.

## 2. Materials and Methods

*Study design*: The study was carried out in a semi-experimental, prospective context, with evaluation at 3, 6 and 10 weeks.

*Sampling method*: The sample consisted of a prospective dynamic cohort of people over 18, admitted for treatment due to a substance abuse disorder and residentially admitted to the therapeutic community. They were recruited by consecutive sampling over a period of 12 months.

The inclusion criteria were: (a) Meeting DSM-5 criteria [29] for substance abuse disorder and the presence of any other mental disorders. (b) Total withdrawal from alcohol and other substances from the beginning of treatment. All patients received a detoxification treatment and were stabilized with medication before they were included in the study. (c) Treatment carried out inside the therapeutic community.

The exclusion criteria were: (a) Patients whose functioning could be altered by factors not specifically related to addictive pathology or mental disorder (severe cognitive impairment, intellectual deficiency or language barrier). (b) Animal-specific phobia.

Initially, 65 subjects were recruited but, after completing the AAT, a final sample of 43 subjects who finished the study was obtained. All patients leaving the study were due to the termination of admission to the therapeutic community, nine of them (40.9%) due to recovery, the rest due to voluntary discharge.

*AAT description*: The AAT was implemented by a therapist who completed a postgraduate diploma in “Animal Assisted Therapies: Foundation and Practice” from the University of Valencia (Spain). The AAT was carried out with one dog, understood as a therapeutic tool, in a group therapy with 10 patients. Regarding the stress of the animal, only one weekly group was made and after each session the dog had a walk, distraction and play session with the therapist. The program consisted of 10 sessions with a duration of about 60 min, carrying out one session per week. Table 1 details the AAT scheme. Patients undertook two types of exercise with the animal. The first sought to create a therapeutic environment suitable for the completion of the second exercise, where patients worked on the designed content in a more specific way with the dog. Table 2 details the contents of each of the sessions.

*Data collection*: was carried out three times during the program with evaluations at Sessions 3, 6 and 10. The clinical instruments used were:

- Ad hoc questionnaire of socio-demographic, psychopathological and health variables;
- The Life Skills Profile questionnaire (LSP) [30]. This questionnaire evaluates aspects of functioning that affect daily activities and the adaptation of people with mental illnesses in the community. It contains 39 items that are grouped into self-care, social-interpersonal behaviour, communication-social contact, non-personal social behaviour and autonomous life;
- Barrat impulsiveness scale (BIS-11) [31]. This instrument is composed of 30 items distributed into three sub-scales: nonplanning, motor and attentional [32]. The total impulsiveness score was also obtained.

*Ethics aspects*: participants signed the relevant informed consent after the six month duration AAT program [33]. This study complies with the principles of the Ethics Committee of the Universidad Católica de Valencia San Vicente Mártir as well as those of the Declaration of Helsinki and the Council of Europe Convention. Confidentiality of the participants and the data was guaranteed according to Organic Law 15/1999 on Personal Data Protection (LOPD).

*Data analysis*: was performed with the SPSS v.21 program. After the exploratory and descriptive analysis, ANOVA of repeated measures was used to assess the differences in the dependent variables between the three time points of evaluation (Sessions 3, 6 and 10).

## 3. Results

In relation to the sociodemographic characteristics obtained, the average age of the patients was 34.4 years (*SD* = 7.4), with a gender distribution of 86% (N = 37) men and the remaining 14% (N = 6) women. Regarding marital status, 46.5% (N = 20) were single, 37.2% (N = 16) divorced/separated, 2.3% (N = 1) had a common-law partner and 14% (N = 6) were married. When evaluating the level of education, the distribution was as follows: 2.3% (N = 1) university graduates, 11.6% (N = 5) without education, 34.9% (N = 15) basic education, 32.6% (N = 14) with secondary education. Of the total sample, 59% (N = 25) were employed prior to treatment and 93% (N = 40) had the support of their family in their treatment.

Regarding variables related to the state of physical health, we found that 7% (N = 3) had a diagnosis of hepatitis C, 4.7% (N = 20) tested positive for hepatitis B and C, and 2.3% (N = 1) had HIV. In the psychopathological variables, we found a percentage of hospital admissions after mental health emergency care of 14.4% (N = 6), 27% (N = 11) with autolytic history and an average of 24.4 years of substance abuse (*SD* = 8.2).

Regarding the substances abused, most of the patients (60.4%; N = 26) were polydrug abusers, cocaine together with alcohol and THC, 13.9% (N = 6) of the patients consumed alcohol, 9.3% (N = 4) heroin and cocaine and the same percentage only cocaine. A total of 4.6% (N = 2) of the patients consumed heroin and 2.3% (N = 1) THC (Hash/Marijuana). In relation to dual pathology, the mental disorders with the highest prevalence were mood disorders at 39.5% (N = 17), followed by Schizophrenia spectrum disorders (25.5%; N = 11) and personality disorders (23.2%, N = 10). A total of 4.6% (N = 2) of the patients suffered from bipolar disorder, and, finally, 6.9% (N = 3) had other mental disorders.

Table 3 shows the results obtained by the participants in the LSP questionnaire, differentiating the various evaluation times. There were statistically significant differences after the AAT intervention in the self-care scales (*F* = 76.09, *p* < 0.001) interpersonal social behaviour (*F* = 68.2, *p* < 0.001), communication (*F* = 45.04, *p* < 0.001), non-personal social behaviour (*F* = 68.73, *p* < 0.001) and autonomous life (*F* = 54.03, *p* < 0.001).

Table 4 shows the impulsiveness scores and the comparisons between the three evaluations performed on the AAT participants. After the implementation of the program, there was a reduction in cognitive impulsiveness, although not statistically significant (*F* = 4.39, *p* = 0.024), and in motor level impulsiveness (*F* = 3.82, *p* = 0.036), planning (*F* = 5.58, *p* < 0.010) and total impulsiveness (*F* = 4.67, *p* < 0.019) compared to the initial values.

## 4. Discussion

The magnitude of the psychological, physical health and social impact of substance abuse disorders, especially when they coexist with a mental illness (dual pathology), demands more research, as well as the design of possible intervention or treatment strategies, with the aim of increasing the quality, reliability and effective management of this reality [34]. Our sample is representative of this impact, because all suffered from dual pathology, with mood disorders being the most comorbid mental illness, and 14.4% having had at least one hospital admission in a mental health unit. These data reflect that substance abuse is closely related to the mental health of the consumer, without forgetting that health deterioration is presented only as a consequence and not as a predisposing factor for substance use [35]. The results obtained on the state of physical health also show a deterioration, with an elevated prevalence of hepatitis C, hepatitis B and HIV infection. Regarding the social impact, the most striking finding was that over half the subjects evaluated were not working, and it was also interesting to note that 93% of our subjects had some type of family support. We consider that this factor may have had some relationship with the psychological consequences for our subjects, namely the possibility of accessing treatment and receiving emotional support [36]. All these repercussions of addiction, and the severity of the addiction itself—polydrug abuse was the norm in our sample—could be the reason for the necessity of a residential setting instead an ambulatory one.

Following the demands of more research, as well as the design of new treatment strategies for patients with dual pathology [37], we have evaluated the viability of implementing an AAT program, with the aim of reducing the psychological, physical health and social consequences of addiction. Specifically, we have evaluated impulsivity in the psychological area and the effectiveness of AAT for life skills for the social area. Regarding the results obtained from the BIS-11 questionnaire, a decrease in impulsiveness was found, in the planning area and as a global factor. From the data obtained, and taking into account other studies [38], it can be concluded that the implementation of AAT resulted in an encouraging improvement in impulsiveness, which is linked to improvement of psychopathology and, specifically, substance abuse disorders themselves. The decrease in impulsiveness in patients reflects the results obtained in research derived from other applications of AAT, such as studies in attention deficit hyperactivity disorders (ADHD), where AAT is applied in a complementary way, obtaining a positive reduction in symptoms and impulsiveness [39,40]. Regarding the improvement in life skills obtained after the implementation of AAT in patients with dual pathology, other studies have demonstrated similar results. In one such study, AAT was shown to improve social skills in a group of young people diagnosed with autism spectrum disorder (ASD), resulting in a decrease in functional problems and reduction in the affective symptoms of the disease [41].

AAT’s effectiveness in patients with dual pathology requires time, as the decrease in impulsiveness was found to occur between the 6th and 10th AAT sessions. It is considered as an appropriate longitudinal approach in our study, to evaluate the predictive power of the intervention related to its global clinical evolution and, as far as possible, in different therapeutic scenarios [42,43]. This conjunction, far from being ambitious, enables a treatment design involving AAT that is applicable to the reality of residential patients [42,44]. After its implementation and effectiveness demonstration, it is proposed that it may be interesting to analyse the possible mechanisms that are linked to a good clinical evolution of patients [45], as well as the shortage of studies regarding the application of AAT in dual pathology [46,47].

The main limitation of the study is the absence of a control group. As semi-experimental study, the possible effect on the results by the residential treatment itself has to be taken into account. Nevertheless, the results obtained seem to follow the line of other studies that denote the usefulness of AAT with minors [48], substance abuse disorders [46,49], ADHD [39], neuro-rehabilitation [47] and ASD [41]. We did not register past dog ownership; it should be considered in future studies as a covariant.

## 5. Conclusions

As has already been demonstrated in other types of patients, with different diseases and in different settings, the authors consider that this study and its results demonstrate that AAT can be implemented in patients with dual pathology in the therapeutic community.

This study demonstrated that AAT, carried out in patients with dual pathology in a residential setting, reduces impulsiveness as well as improving different life skills. Nevertheless, it is worth mentioning that it would be wrong to use AAT as the sole intervention for improving symptoms. AAT should be used as a complement to approaches that use cognitive-behavioural, pharmacological or other types of therapeutic interventions.

## Figures and Tables

**Table 1:** Scheme of the sessions.

Schedule	Objective	Content
Presentation	Greeting	Introduction of the dog
Warm-Up exercise	Attention	Uniting patient and dog
Specific exercises	Therapy	Aims of the programme
Shared time	Expression	Cognitive-behavioural
Parting	Finish	Farewell

**Table 2:** Animal-assisted therapy sessions content.

Session Number	Warm-Up Exercise	Specific Exercise
First session	Hello friend	Sit and lie down
Second session	May I introduce my friend	Quiet and come here
Third session	Petting	Slalom between the legs
Fourth session	Brushing the dog	Jumping over obstacles
Fifth session	Dressing the dog	Walking
Sixth session	Hello	Sleep
Seventh session	What about if I scratch you?	Dog gymkhana
Eighth session	Look at me	Hide
Ninth session	Give me a paw	Childhood pets
Tenth session	The dog greets the group	Hand delivery

**Table 3:** Results of everyday life skills (LSP).

Variables		Time of Test Session			F	Sig.	Differences Significant	ES	Power
		3	6	10					
Self-care	M	18.70	26.36	31.61	76.09	<0.001 ***	3 < 6 and 6 < 10	0.95	1
	SD	7.10	7.05	6.42					
Interpersonal social behaviour	M	18.06	24.26	29.28	68.20	<0.001 ***	3 < 6 and 6 < 10	0.70	1
	SD	6.27	6.04	5.43					
Communication	M	11.05	14.06	17.31	45.04	<0.001 ***	3 > 6	0.60	1
	SD	4.48	3.47	3.77					
Non-personal social behaviour	M	11.10	14.76	17.51	68.73	<0.001 ***	3 < 6	0.70	1
	SD	3.62	3.68	3.44					
Autonomous life	M	13.51	16.65	20.25	54.03	<0.001 ***	3 < 6 and 6 < 10	0.65	1
	SD	4.18	3.99	3.96					

Note: *** p < 0.001; ES: effect size.

**Table 4:** Barratt Impulsiveness scale (BIS-11) results.

Variables		Time of Test Session			F	Sig.	Differences Significant	ES	Power
		3	6	10					
Cognitive impulsiveness	M	14.42	15.26	12.23	4.39	0.024 *	Not significant	0.26	0.70
	SD	6.27	4.41	5.42					
Motor impulsiveness	M	23.65	24.46	19.76	3.82	0.036 *	Not significant	0.24	0.63
	SD	9.46	6.72	8.81					
Planning impulsiveness	M	40.69	42.11	32.65	5.58	0.010 *	10 < 6	0.31	0.80
	SD	13.55	8.92	15.79					
Total impulsiveness	M	78.76	81.92	65.23	4.67	0.019 *	10 < 6	0.28	0.73

Note: * p < 0.05; ES: effect size.
